# Host–device interactions: exposure of lung epithelial cells and fibroblasts to nickel, titanium, or nitinol affect proliferation, reactive oxygen species production, and cellular signaling

**DOI:** 10.1007/s10856-023-06742-2

**Published:** 2023-07-24

**Authors:** Simon D. Pouwels, Alina Sigaeva, Shanna de Boer, Ilse A. Eichhorn, Lisanne Koll, Jeroen Kuipers, Romana Schirhagl, Irene H. Heijink, Janette K. Burgess, Dirk-Jan Slebos

**Affiliations:** 1 Department of Pulmonary Diseases, University of Groningen, University Medical Center Groningen, Hanzeplein 1, 9713 GZ Groningen, The Netherlands; 2 Department of Pathology and Medical Biology, University of Groningen, University Medical Center Groningen, Hanzeplein 1, 9713 GZ Groningen, The Netherlands; 3 University of Groningen, University Medical Center Groningen, Groningen Research Institute for Asthma and COPD (GRIAC), Hanzeplein 1, 9713 GZ Groningen, The Netherlands; 4 Department of Biomedical Engineering, University of Groningen, University Medical Center Groningen, Antonius Deusinglaan 1, 9713 AW Groningen, The Netherlands; 5 Department of Biomedical Sciences of Cells and Systems, University of Groningen, University Medical Center Groningen, Groningen, The Netherlands

## Abstract

Endoscopic implantation of medical devices for the treatment of lung diseases, including airway stents, unidirectional valves and coils, is readily used to treat central airway disease and emphysema. However, granulation and fibrotic tissue formation impairs treatment effectiveness. To date little is known about the interaction between implanted devices, often made from metals, such as nickel, titanium or nitinol, and cells in the airways. Here, we study the response of lung epithelial cells and fibroblasts to implant device materials. The adhesion and proliferation of bronchial epithelial cells and lung fibroblasts upon exposure to 10 × 3 × 1 mm pieces of nickel, titanium or nitinol is examined using light and scanning electron microscopy. Pro-inflammatory cytokine mRNA expression and release, signaling kinase activity and intracellular free radical production are assessed. Nitinol, and to a lesser extent nickel and titanium, surfaces support the attachment and growth of lung epithelial cells. Nitinol induces a rapid and significant alteration of kinase activity. Cells directly exposed to nickel or titanium produce free radicals, but those exposed to nitinol do not. The response of lung epithelial cells and fibroblasts depends on the metal type to which they are exposed. Nitinol induces cellular surface growth and the induction of kinase activity, while exposure of lung epithelial cells to nickel and titanium induces free radical production, but nitinol does not.

**Graphical Abstract:**

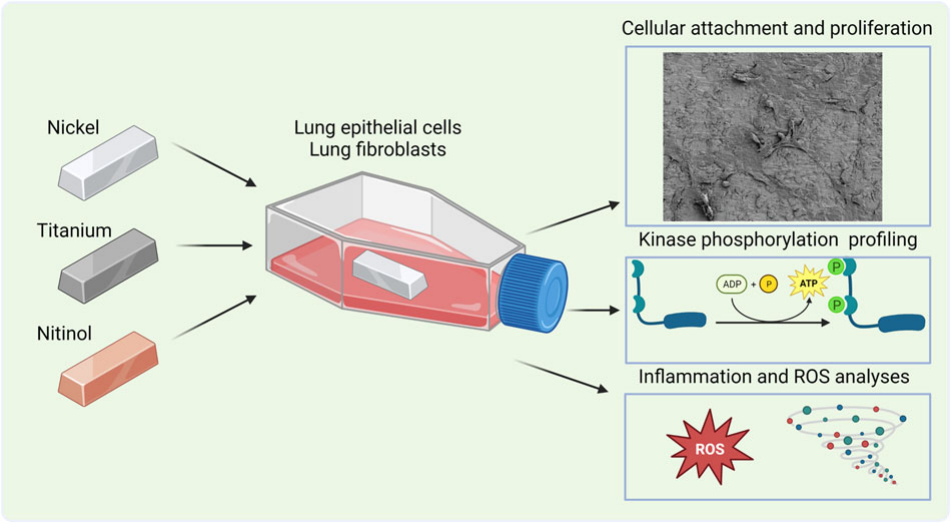

## Introduction

Medical devices that can be endoscopically implanted see a rapid growth in usage for the treatment of both malignant and benign lung diseases. These lung implantable devices include endobronchial one-way valves, stents and coils [1]. For some patients with severe chronic obstructive pulmonary disease the implantation of medical devices is the only treatment option to prevent airway obstruction and to reduce the trapping of air in emphysematous lung tissue [2]. Most of these devices are at least partly made out of nitinol, an alloy of equal amounts of nickel and titanium that is widely used for implantable devices due to its unique properties, such as shape memory and superelasticity [3]. These properties mean that nitinol devices can be folded into the cramped space of a catheter, while immediately returning to their original shape upon release. Airway stents made from nitinol, either coated with silicon or uncoated, have been regularly used clinically for many years to reduce central airway obstructions [4]. A more recently approved clinical treatment option for severe emphysema patients is the treatment with endobronchial valves; one-way valves made out of a nitinol mesh covered in silicon [5]. Additionally, more experimental lung implantable devices such as endobronchial coils implanted in the sub-segmental airways to improve lung function in severe emphysema patients are also made out of nitinol [6].

Treatments with nitinol implantable devices have proven to be successful, leading to significant improvements of lung function, exercise capacity and overall quality of life [2]. Nevertheless, in many patients the effectiveness of the treatment is lost over time, often caused by the formation of granulation tissue around the implanted device. Granulation tissue is a complex tissue consisting of large accumulations of inflammatory cells, newly formed connective tissue and even small blood vessels [7]. After the implantation of endobronchial valves 41% of patients required a revision bronchoscopy within 2 years, of which 53% showed severe granulation tissue formation causing loss of function of the endobronchial valves [2]. Moreover, in central benign and malignant airway disease, up to 57% of patients with implanted nitinol stents experienced loss of stent-function due to the formation of granulation tissue within the stent [8–10]. To date, the exact cellular and molecular mechanisms of how and when granulation tissue formation occurs following the implantation of lung implantable devices remains largely unknown. Several reasons have been suggested in literature, including increased bacterial colonization, genetic susceptibility and friction or pressure-related damage induced by the combination of the shape and size of the implant with the constant motion of lung tissue during breathing and coughing [7]. It is likely that a combination of intrinsic and extrinsic factors determines whether a patient develops granulation tissue formation in response to nitinol implantation. The foreign body response, the inflammatory reaction preceding the formation of granulation tissue, is likely initiated by the interaction of lung structural cells, such as epithelial cells and fibroblasts, with the implantable device material. Damaged, stressed and activated lung epithelial cells and fibroblasts can release pro-inflammatory as well as pro-fibrotic cytokines, reactive oxygen species and endogenous danger signals that can attract cells of the innate immune systems, e.g., neutrophils and macrophages, which ultimately may contribute to the formation of granulation tissue.

There are some data available indicating that the surface properties of nitinol can affect the proliferation rates and cellular attachment capabilities of osteoblasts and endothelial cells [11], but no data is available on the effects of nitinol on lung structural cells. Furthermore, it is well-established that metals often used for implantable devices, such as nickel, and to a lesser extent titanium, can induce inflammation, the release of free radicals and cell death [12–15], but there no data available on the cellular responses of lung epithelial cells and fibroblasts in response to nitinol.

There is an urgent need for the development of novel devices to keep the airways open and to deflate hyperinflated lung areas without causing severe side-effects, such as the formation of granulation and fibrotic tissue. Several different materials can be used for implantable devices, such as stainless steel, titanium, nitinol and silicon-based materials. For devices implanted in the lungs nitinol often has been chosen as the base material, partly due to the unique material properties of nitinol, such as shape-memory and hyper-elasticity. However, it is still unknown, if and how nitinol is contributing to the development of granulation and fibrotic tissue upon implantation. Therefore, in the current study the first steps were taken towards unraveling the molecular responses induced by nitinol and its core components, nickel and titanium in lung epithelial cells and fibroblasts. Here, a comparison was made between the effects of nickel, titanium and nitinol, with respect to cell viability, growth and attachment, as well as inflammatory marker release and free radical production, in structural lung cells. The main aim of this study was to assess cellular and molecular responses of lung epithelial cells and fibroblasts that were either in direct contact with, or in the close proximity of metal. This will provide new insights into processes involved in granulation tissue formation around lung implantable devices.

## Materials and methods

### Cell culture

The human bronchial epithelial cell line BEAS-2B was cultured in RMPI-1640 growth medium (Lonza, Basel, Switzerland) supplemented with 10% (v/v) fetal bovine serum (FBS), 50 μg/mL streptomycin and 50 U/mL penicillin at 37 °C with 5% CO_2_. Fetal human lung MRC5 fibroblasts [16] were cultured in Dulbecco’s Modified Eagle Medium (Lonza, Basel, Switzerland) supplemented with 4.5 g/L glucose, 10% (v/v) FBS, 50 μg/mL streptomycin, 50 U/mL penicillin, and 1% (v/v) GlutaMAX (Gibco, Waltham, MA) at 37 °C with 5% CO_2_.

To study the cellular behavior upon exposure to metals, 30,000 BEAS-2B cells/well or 40,000 MCR-5 fibroblasts/well of a 24-well plate, were seeded and grown until ~90% confluent at day three. A 10 × 3 × 1 mm piece of nickel, titanium or nitinol (generous gift from FreeFlowMedical, Freemont, CA) was placed in the center of the well either just prior to seeding or immediately after a confluent monolayer was formed. Afterwards, cells were visually monitored for 72 h. At the same time, cells were collected for RNA isolation and cell-free supernatants were harvested. Images were obtained daily using a light microscope (Leica MC120 HD, Wetzlar, Germany).

### Gene expression analysis, cytokine, and dsDNA release and kinase phosphorylation

For quantitative real-time polymerase chain reaction (qRT-PCR) analyses, cells were lysed in 500 µL TRIzol (Ambion/Life technologies, Oudeschoot, The Netherlands) and stored at −20 °C until further usage. Afterwards, RNA was isolated as described before [17], and converted into cDNA using the RevertAid First Strand cDNA Synthesis Kit (Thermo Fisher Scientific, Waltham, MA) according to the manufacturer’s protocol. Next, the quantitative mRNA expression was assessed using TaqMan gene expression assays, using universal TaqMan master mix (Promega, Madison, WI) and an ABI 7900HT Real Time Thermo Cycler 384 (Applied Biosystems, Waltham, MA) according to the manufacturer’s protocol. The following primer/probe sets specific for target genes were used (Thermo Fisher Scientific, Waltham, MA): *IL6* (Hs00174131_m1), *CXCL8* (Hs00174103_m1), *MYD88* (Hs00182082_m1), *TGFB1* (Hs00998133_m1), and *B2M* (Hs00187842_m) as reference gene.

Secreted levels of the pro-inflammatory cytokine CXCL8 were measured in cell-free supernatants using a sandwich Enzyme Linked Immunosorbent Assay (ELISA) specific for CXCL8 (R&D Systems, Minneapolis, MN; Human IL-8/CXCL8 DuoSet ELISA: DY208). Optical density was measured at 450 nm using a microplate reader (EL-800, BioTek, Winooski, VT). Extracellular levels of the damage associated molecular pattern dsDNA were measured using the Quant-iT™ PicoGreen® dsDNA Assay Kit (ThermoFisher Scientific, Waltham, MA: P11496). Fluorescence was measured at 480 nm (extinction)/530 nm (emission) using a fluorescent microplate reader (Clariostar plus, BMG Labtech, Ortenberg, Germany).

To detect the relative levels of phosphorylation of 37 kinases at specific sites, as well as the expression of 2 substrate proteins, a Proteome Profiler Human Phospho-Kinase Array Kit (ARY003C, R&D Systems, Minneapolis, MN) was used. Per analysis 600 μg of protein was isolated, measured using the protein assay kit (Pierce BCA Protein Assay Kit, Thermo Fisher Scientific, Waltham, MA), and directly used. Chemiluminescence was detected using the ChemiDoc MP imaging system (Bio-Rad, CA).

### Scanning electron microscopy

A 10 × 3 × 1 mm piece of nickel, titanium or nitinol with BEAS-2B cells cultured on its surface were fixed for Scanning Electron Microscopy (SEM) in 2% (v/v) glutaraldehyde in 0.1 M cacodylate buffer, and stored until usage at 4 °C. Next, the samples were incubated for 1 h in 1% (v/v) osmium tetroxide (OsO_4_) in 0.1 M cacodylate buffer and afterwards the metals were washed three times with Milli-Q ultrapure water. The samples were dehydrated in 30%, 50%, and 70% ethanol, 10 min each, followed by four times dehydration in 100% ethanol for 10, 20, and two times 30 min respectively. Lastly, the samples were dried by incubating for 10 min in 1:1 100% ethanol/tetramethyl silane (Acros, Geel, Belgium) and for 15 min in pure tetramethyl silane followed by air-drying.

After sample preparation, the pieces of metal were placed on a carbon holder and subsequently sputter coated (Leica EM SCD050 and QSG100, Wetzlar, Germany) with 4 nm palladium/gold.

Samples were imaged with a Zeiss Supra 55 SEM (Oberkochen, Germany) using a secondary electron detector at an acceleration tension of 3.0 kilovolt (kV) with 30 µm aperture at 7.2 mm working distance.

### Relaxometry

For the detection of free radicals produced by BEAS-2B cells after exposure to titanium, nickel and nitinol, fluorescent nanodiamonds (FNDs) containing nitrogen-vacancy (NV) centers for quantum sensing with a hydrodynamic diameter of 70 nm were purchased from Adamas Nanotechnology (Raleigh, North Carolina, USA). These particles are produced by the manufacturer by high pressure high temperature synthesis followed by irradiation with 3 MeV electrons at a fluence of 5 × 10^19^ e/cm^2^ and contain around 500 NV centers per particle [18]. The particles are widely used in the field and have been extensively characterized in literature [19, 20]. To investigate T_1_ relaxation of FNDs internalized by BEAS-2B cells, FNDs were first mixed with FBS and diluted further in serum-free RPMI medium (final FND concentration: 1 µg/mL) to prevent particle aggregation. Epithelial BEAS-2B cells were cultured in 35-mm glass bottom Petri dishes until confluent monolayers were formed. Prior to the relaxometry measurements, the cell culture medium in the dish was replaced with the FND solution and cells were incubated with FNDs for 2 h at 37 °C, 5% CO_2_. Afterwards, a piece of nickel, titanium, or nitinol was placed in the center of the Petri dish and incubated for 4 or 48 h. Relaxation T_1_ measurements were performed on a homemade magnetometer containing a green laser (Torus 532 nm, Laser Quantum, Telford, UK) attenuated to 70 µW (at the position of the sample measured in continuous illumination) which was focused by a microscope objective (Olympus UPLSAPO 100XO, Oil immersion, NA 1.40, Tokyo, Japan) on the sample. Photoluminescence was collected by the same objective, filtered by a 600 nm longpass dichroic mirror and dielectric filter to send photon counting avalanche photodiode (Excelitas Technologies SPCM-AQRH, Waltham, Massachusetts, USA) through a confocal pinhole. Live cells were imaged by scanning the beam over the sample, both cells and nanodiamonds were visible. The intracellular localization of every particle was confirmed by confocal microscopy. After localizing a single internalized FND, the T_1_ measurement was performed, as described previously [21–23]. As T_1_ relaxation of NV centers happens faster in presence of higher concentrations of paramagnetic species (such as free radicals), shorter T_1_ times correspond to a higher radical load inside the cell. The movement of FNDs during the measurements was tracked every 3 s by making a confocal scan over a 2 × 2 µm area and using the Gaussian fit to find the new position of the FND. At least 33 T_1_ curves from different FNDs were recorded for each experimental condition (different timepoints, materials, cells being or not being in direct contact with the metal piece).

### Statistics

All statistical analyses were performed using GraphPad Prism 5 (GraphPad Software Inc. San Diego, CA) All experiments were performed six times unless stated differently. Statistical differences between metals were determined using a Mann-Whitney *U* test, where *p* < 0.05 was considered statistically significant.

## Results

### Viability and growth of lung epithelial cells and fibroblasts is affected by exposure to metal

**Fig. 1:**
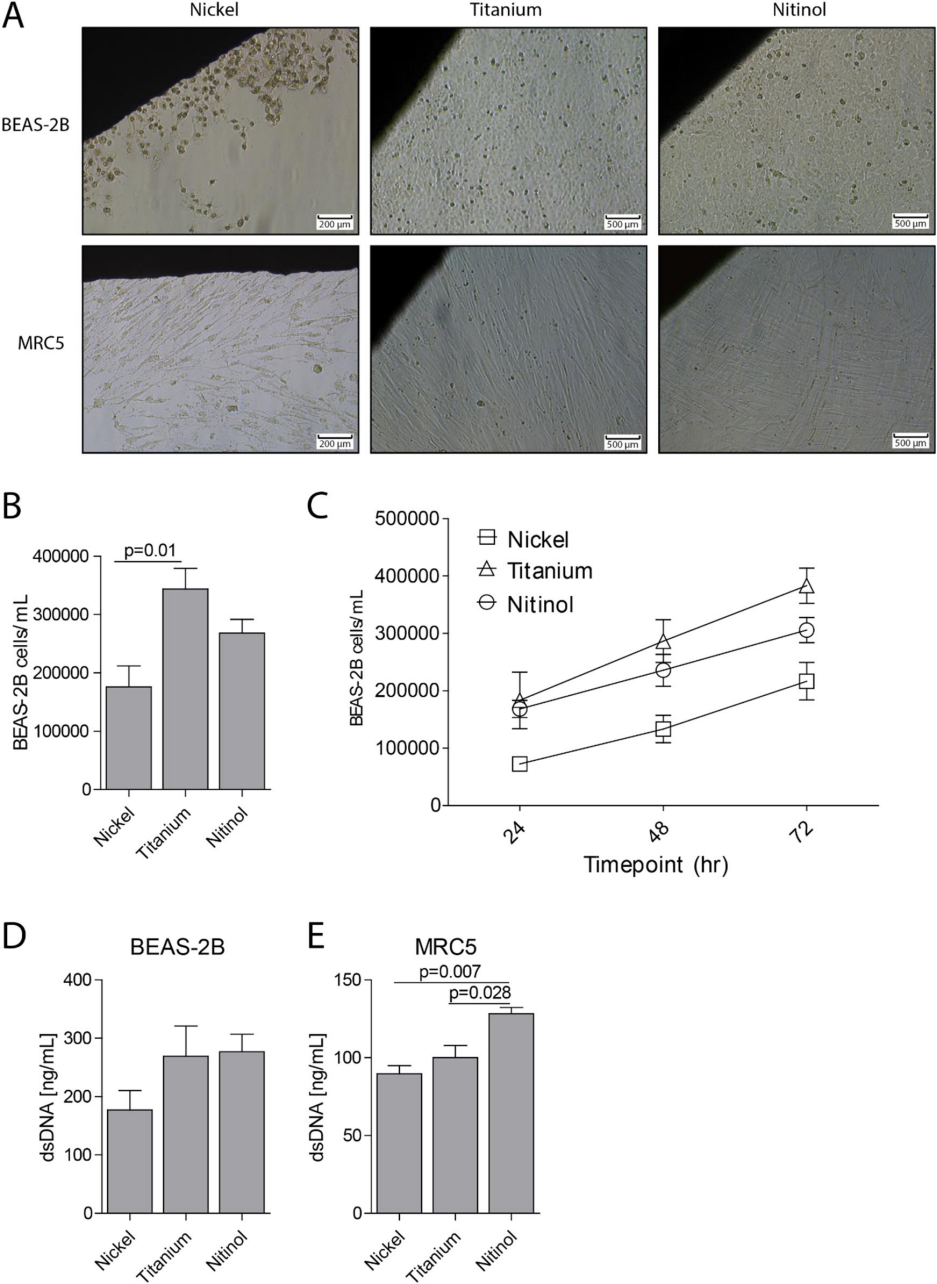
Nickel affects the growth and viability of lung epithelial cells. Human bronchial epithelial cells (BEAS-2B) and fibroblasts (MRC5) were grown in the presence of 10 × 3 × 1 mm pieces of nickel, titanium or nitinol. **A** Cell viability around the edges of the metal was visually inspected using light microscopy after 72 h of culture. **B** the number of BEAS-2B cells present in the well after 72 hours of culture in the presence of metal. **C** The number of BEAS-2B cells present in the well after 72 hours as presented in panel B, with the addition of the number of cells after 24 and 48 h of culture in the presence of metal. The concentration of dsDNA was measured in the supernatant of (**D**) BEAS-2B or (**E**) MRC5 cells as a measure of necrotic cell death after culturing for 72 h in the presence of metal. The scale bar represents 200 µm for nickel and 500 µm for titanium and nitinol as indicated. All experiments were performed six times. Data is shown as mean ± SEM. Significance was tested between the different metals using a Mann-Whitney *U* test, where *p* < 0.05 is considered as statistically significant. The *p* values of significant results are indicated above a horizontal line between the tested conditions

Several lung implantable devices, such as stents, coils and valves, are made out of the super-elastic metal nitinol, an alloy made from titanium and nickel. In order to investigate the effects of pieces of metal on lung epithelial cells and fibroblasts, human bronchial epithelial cells (BEAS-2B) and fetal human lung fibroblasts (MRC5) were cultured in the presence of a 10 × 3 × 1 mm piece of nickel, titanium or nitinol. Both BEAS2-B and MRC5 cells were able to form a confluent monolayer in the presence of titanium and nitinol within 72 h without any visual signs of aberrant cell viability or extensive cell death (Fig. 1A). However, upon culture in the presence of nickel, BEAS-2B cells largely refrained from growing in the close vicinity of the nickel piece and excessive patches of dead cells were observed throughout the well (Fig. 1A). Lung fibroblast MRC5 cells were slightly more resistant towards nickel, although the cell density near the edge of the nickel piece was also reduced in MRC5 cells (Fig. 1A). Next, BEAS-2B cell growth was assessed by measuring the number of cells daily for 3 days. After 3 days the number of cells that grew in the presence of nickel was significantly lower compared to the number of cells grown in the presence of titanium. (Fig. 1B). At every timepoint that was assessed the number of cells that grew in the presence of Nickel was lower compared to cells that grew in the presence of Titanium or nitinol, indicating that nickel suppresses cell growth (Fig. 1C). To assess whether cells that grow in proximity of metal go into necrosis, the levels of the necrotic cell death marker dsDNA were measured in cell free supernatant. Cells grown in the presence of different metals did not show differences in the levels of dsDNA in supernatant of BEAS-2B cells, while a significant increase in the levels of extracellular dsDNA was shown for MRC5 cells that were grown in the presence of nitinol (Fig. 1D, E). Together, both bronchial epithelial cells as well as lung fibroblasts display reduced cell growth in close proximity to nickel. Furthermore, fibroblasts grown in the presence of nitinol secreted significantly more dsDNA, suggesting that nitinol induces cell death in fibroblasts.

### Nitinol allows the growth of viable bronchial epithelial cells on its surface

**Fig. 2:**
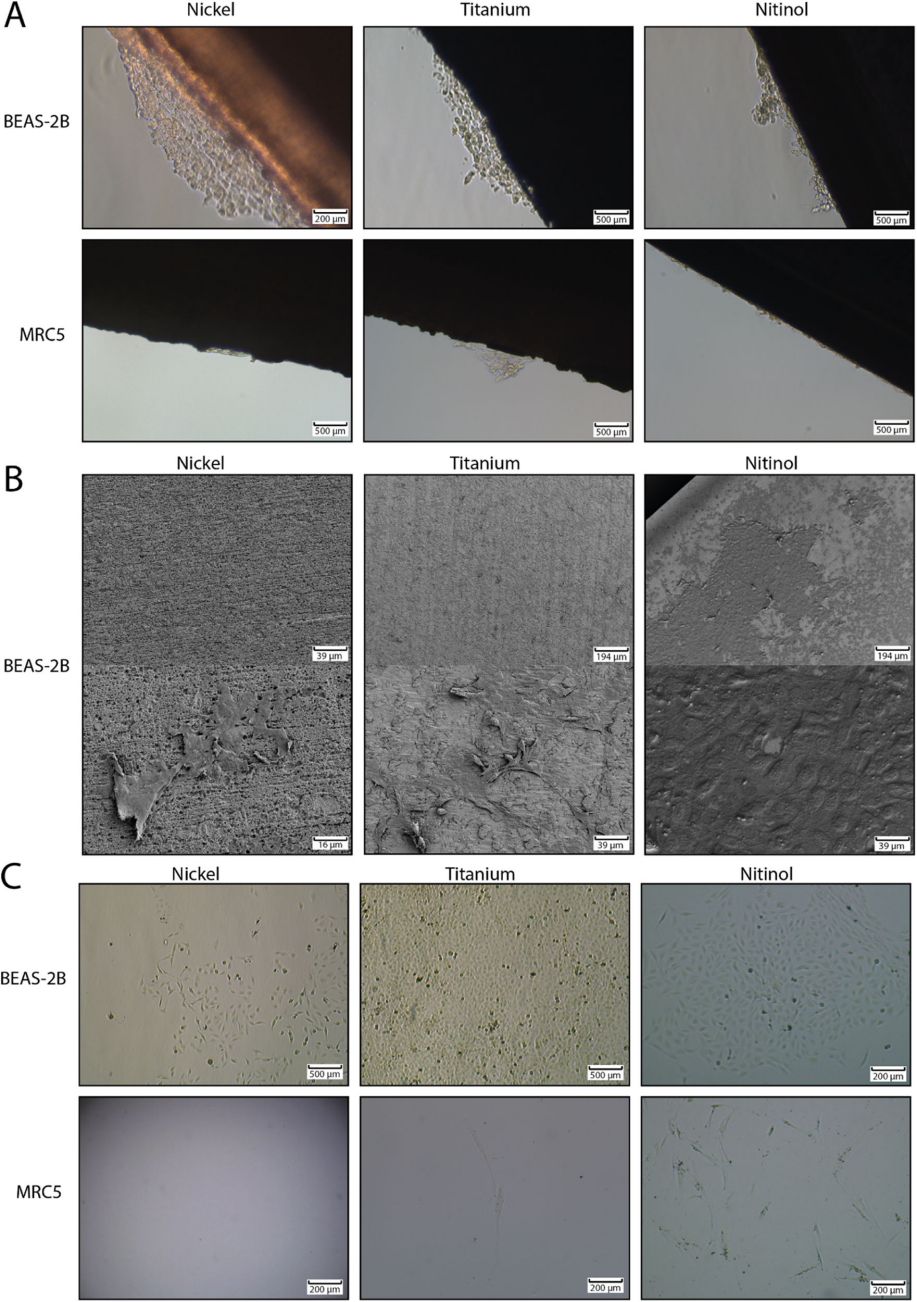
Bronchial epithelial cells grow on the surface of metal. Human bronchial epithelial cells (BEAS-2B) and fibroblasts (MRC5) were grown in the presence of 10 × 3 × 1 mm pieces of nickel, titanium, or nitinol. Afterwards the piece of metal was removed from the well and rinsed with PBS. **A** Cellular attachment to the side of the metal pieces was inspected using light microscopy. **B** Cellular attachment of BEAS-2B cells to the surface of the metal was confirmed using scanning electron microscopy. **C** Viability of the cells attached to the surface of metals was investigated by removing the cells of the rinsed metal pieces using Trypsin. Next, the cells isolated from the surface of the metal pieces were cultured for 72 h before being visually inspected for viable cell growth using light microscopy. All photos are a representation of at least three individual experiments

**Fig. 3:**
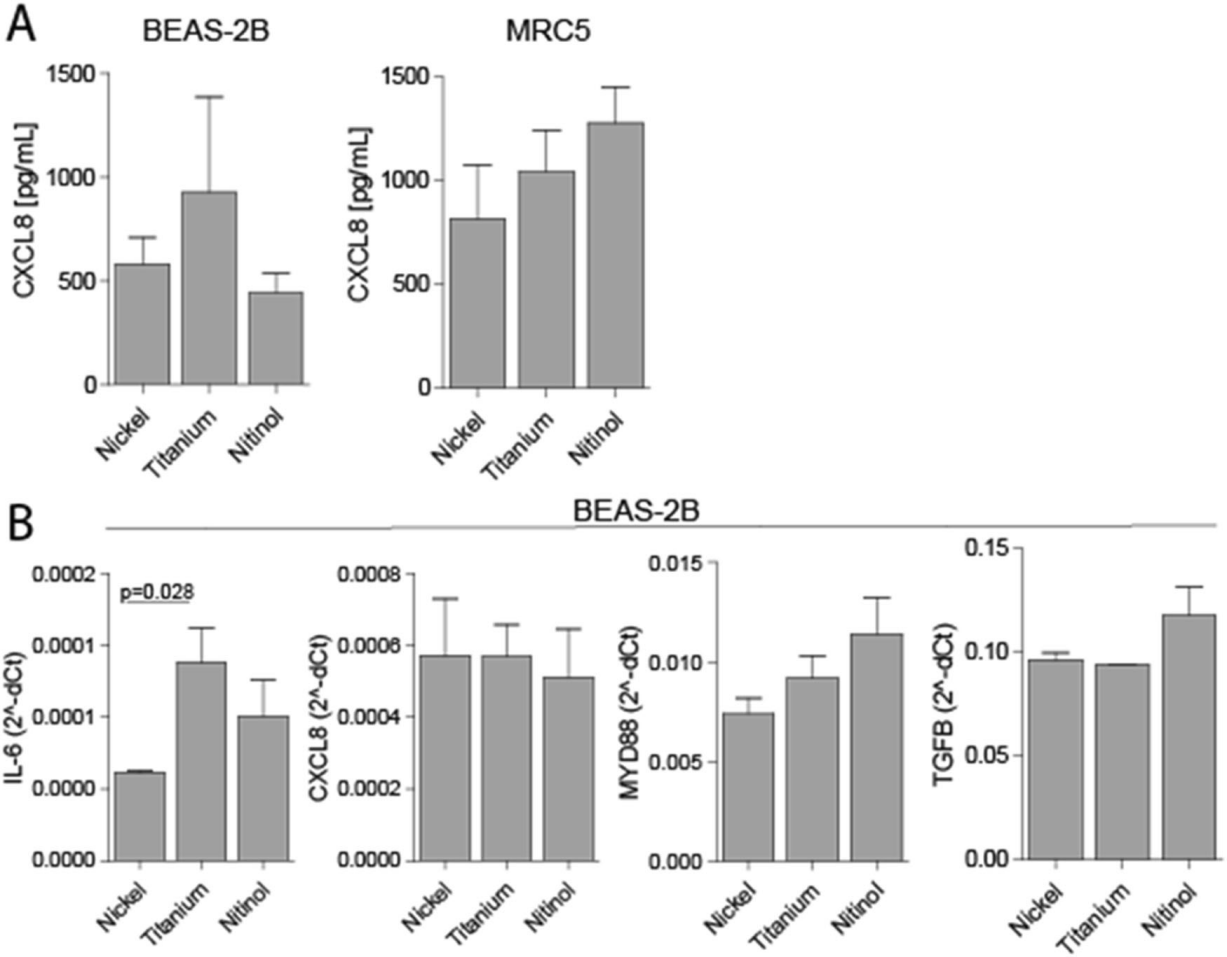
Nitinol does not induce a larger pro-inflammatory response in lung epithelial cells and fibroblasts compared to nickel or titanium. **A** Release of the pro-inflammatory cytokine CXCL8 was measured in the supernatant of BEAS-2B and MRC5 cells following exposure of a mono-layer of cells to a 10 × 3 × 1 mm piece of nickel, titanium or nitinol for 72 h. **B** Expression of pro-inflammatory (IL-6, CXCL8 and MYD88) and pro-fibrotic (TGFB) genes was measured using qRT-PCR in BEAS-2B cells following exposure of a mono-layer of cells to a 10 × 3 × 1 mm piece of nickel, titanium or nitinol for 72 h. All experiments were performed six times. Data is shown as mean ± SEM. Significance between the different metals was tested using a Mann-Whitney *U* test, where *p* < 0.05 is considered as statistically significant. The *p* values of significant results are indicated above a horizontal line between the tested conditions

For most lung implantable devices, cellular encapsulation, cellular attachment and cellular overgrowth are adverse effects, that should be kept to a minimum. Here, it was assessed whether and to which extent BEAS-2B cells and MRC5 fibroblasts were able to grow onto the surface of pieces of nickel, titanium and nitinol. Both BEAS-2B and MRC5 cells were cultured in the presence of nickel, titanium or nitinol for 72 h. Afterwards, the pieces of metal were removed from the wells plate, rinsed with PBS and placed in fresh PBS. Patches of BEAS-2B cells were found attached to the sides of the metal pieces for all three metal types (Fig. 2A). For MRC5 cells, only a few individual cells were found attached to the sides of the metal pieces. Therefore, cellular attachment was only assessed using scanning electron microscopy for BEAS2B cells. Here, it was confirmed that BEAS-2B cells were attached and growing on metal surfaces (Fig. 2B). On a piece of nitinol an almost confluent layer of cells formed on the surface. In order to confirm that the cells that were observed on the surface of the metal pieces were viable, rather than cellular debris passively attached to the metal surface, BEAS-2B and MRC5 cells were trypsinized from the rinsed pieces of metal. The cells isolated from the pieces of metal were cultured in a new plate for 72 h. It was found that BEAS-2B cells isolated from titanium and nitinol subsequently formed a confluent mono-layer, confirming that the isolated cells were viable and able to proliferate (Fig. 3C). Only limited numbers of BEAS-2B cells grew from the cells isolated from nickel, illustrating the less viable condition of these cells. From the MRC5 cells isolated from the metal pieces only a few cells survived for 72 h, further indicating that bronchial epithelial cells are more capable of growing on the surface of metal compared to fibroblasts.

### Exposure of lung epithelial cells to nitinol does not induce a larger pro-inflammatory response compared to nickel or titanium

Next, it was assessed whether exposure of lung epithelial cells and fibroblasts to a piece of metal induced a pro-inflammatory or fibrotic response. First, the levels of the pro-inflammatory cytokine CXCL8 were measured in supernatant from BEAS-2B or MRC5 cells, which were not different between cells exposed to nickel, titanium or nitinol (Fig. 3A). As limited effects of metal on cell growth, cell attachment and pro-inflammatory cytokine release were observed for MRC5 cells, the in depth analyses were only performed using BEAS-2B cells.

The gene expression levels of the pro-inflammatory genes *IL-6*, *CXCL8* and *MYD88*, and the pro-fibrotic gene *TGFB* were measured in BEAS-2B cells grown in the presence of nickel, titanium or nitinol for 72 h. The gene expression levels of *IL-6* were increased in BEAS-2B cells exposed to titanium compared to cells exposed to nickel (Fig. 3B). Furthermore, no significant differences in pro-inflammatory or pro-fibrotic gene expression were observed between cells exposed to pieces of different metals.

### Nitinol strongly alters the kinase activity profile in bronchial epithelial cells

**Fig. 4:**
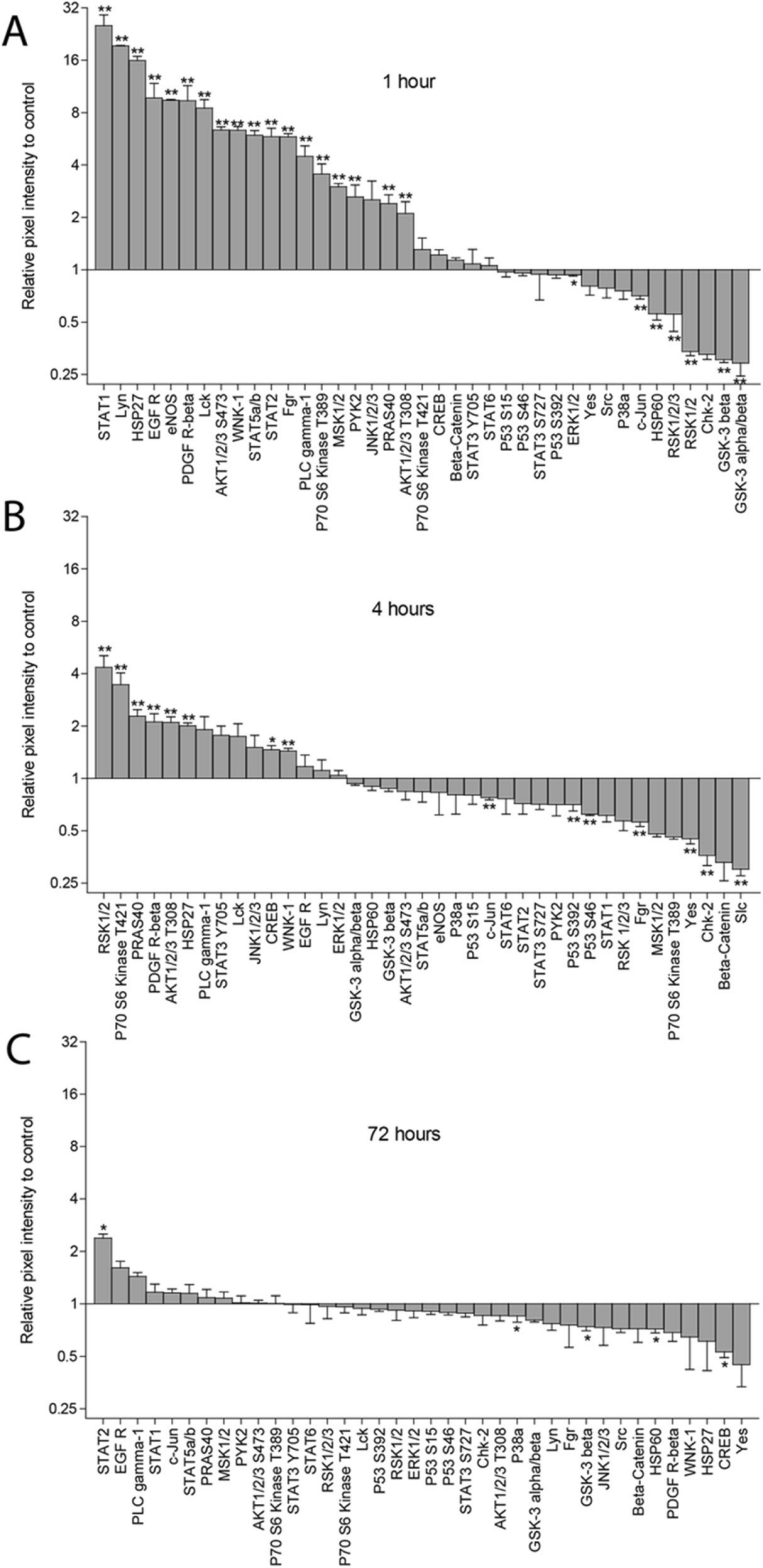
Nitinol affects the phosphorylation profile of kinases and their protein substrates in BEAS-2B cells. A mono-layer of BEAS-2B cells was exposed to a 10 × 3 × 1 mm piece of nitinol for **A** 1, **B** 4, or **C** 72 h. The expression of 39 phosphorylated kinases and their protein substrates was assessed using the Human Phospho-Kinase Antibody Array. The following proteins and kinases with specific phosphorylation sites, between brackets, was assessed: Akt 1/2/3 (S473), Akt 1/2/3 (T308), beta-Catenin, Chk-2 (T68), c-Jun (S63), CREB (S133), EGF R (Y1086), eNOS (S1177), ERK1/2 (T202/Y204, T185/Y187), Fgr (Y412), GSK-3 alpha/beta (S21/S9), p70 S6 Kinase (T421/S424), GSK-3 beta (S9), HSP27 (S78/S82), HSP60, JNK 1/2/3 (T183/Y185, T221/Y223), Lck (Y394), Lyn (Y397), MSK1/2 (S376/S360), p38 alpha (T180/Y182), p53 (S15), p53 (S392), p53 (S46), P70 S6 Kinase (T389), PDGF R beta (Y751), PLC gamma-1 (Y783), PRAS40 (T246), Pyk2 (Y402), RSK1/2 (S221/S227), RSK1/2/3 (S380/S386/S377), Src (Y419), STAT1 (Y701), STAT2 (Y689), STAT3 (S727), STAT3 (Y705), STAT5a/b (Y699), STAT6 (Y641), WNK-1 (T60), Yes (Y426). The data show the relative difference in pixel intensity between BEAS-2B cells exposed to nitinol and control BEAS-2B cells not exposed to metal. All experiments were performed in duplicate, six times. Data are shown as mean ± SEM. Significance was tested between nitinol-exposed and non-exposed BEAS-2B cells using a Mann-Whitney *U* test. Significant differences are indicates using stars for each kinase, * = *p* < 0.05, ** = *p* < 0.01

Although no strong pro-inflammatory responses were found upon exposure of BEAS-2B cells to a piece of nitinol, specific cellular signaling pathways may be more or less active upon exposure to nitinol. The expression of 37 phosphorylated kinases and 2 substrate proteins was assessed in a mono-layer of BEAS-2B cells, either exposed to a piece of nitinol or not exposed to metal for 1, 4, and 72 h. After one hour, strong alterations in kinase activity were observed, with 18 kinases displaying increased levels of phosphorylation at specific activity sites and 6 phosphorylated kinases and Heat Shock protein 60 (HSP60) showing decreased expression (Fig. 4A). Signal transducer and activator of transcription 1 (STAT1) showed the highest increase in expression with 30 times increased expression. After 4 h of exposing BEAS-2B cells to a piece of nitinol, a similar pattern was observed. However, the magnitude of the response was reduced, with an increase in the expression of eight phosphorylated kinases, and a decrease in the expression of seven phosphorylated kinases (Fig. 4B). After 72 h the effect of nitinol on kinase activity had mostly been extinguished, with only the phosphorylation of STAT2 still being increased, while the expression of HSP60 and the phosphorylation of P38a, GSK-3 beta, and CREB were decreased (Fig. 3C). Together, this shows that exposure to a piece of nitinol strongly, and temporarily affects the kinase activity profile of BEAS-2B cells.

### Direct exposure of bronchial epithelial cells to nitinol induces less free radical production compared to other metals

**Fig. 5:**
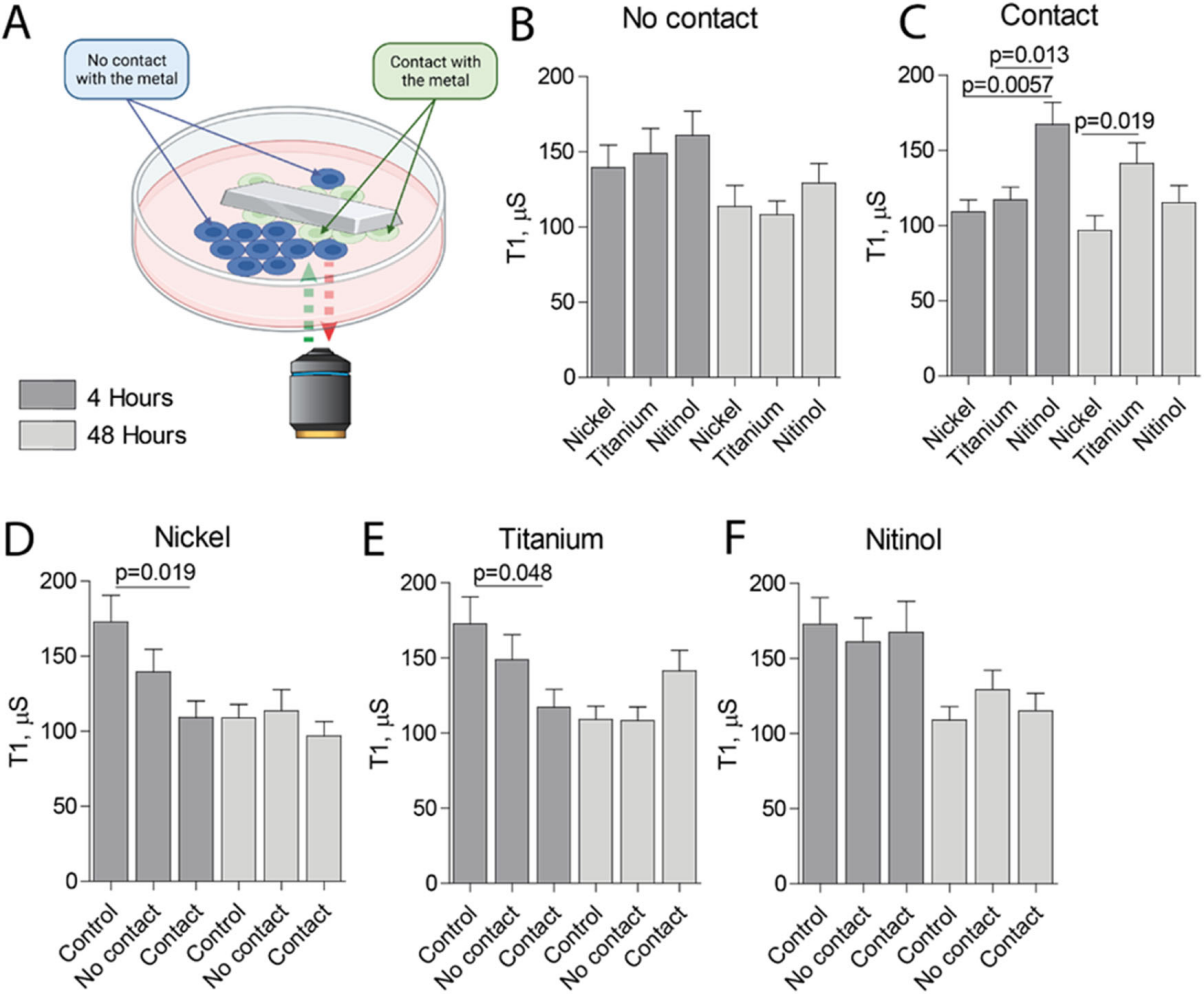
Nitinol induces less free radical production in BEAS-2B cells compared to titanium and nickel. Free radical production was measured using the T_1_ relaxometry mode of diamond magnetometry, where lower T_1_ values represent an increase in free radical production. A confluent layer of human bronchial epithelial cells (BEAS-2B) cells was incubated with 1 µg/mL fluorescent nanodiamonds for 2 h. Afterwards, cells were exposed to a 10 × 3 × 1 mm piece of nickel, titanium or nitinol for 4 or 48 h. **A** T_1_ free radical measurements were performed in cells directly or indirectly exposed to metal. **B** T_1_ measurements in BEAS-2B cells indirectly exposed to nickel, titanium or nitinol. **C** T_1_ measurements in BEAS-2B cells directly exposed to nickel, titanium or nitinol. In order to compare the T_1_ measurements between cells in direct or indirect contact with metal, the data presented in (**B**) and (**C**) is presented per metal type, supplemented with the no-metal control. **D** T_1_ measurements in BEAS-2B cells without metal exposure, directly or indirectly exposed to nickel. **E** T_1_ measurements in BEAS-2B cells without metal exposure, directly or indirectly exposed to titanium. **F** T_1_ measurements in BEAS-2B cells without metal exposure, directly or indirectly exposed to nitinol. The data represents T_1_ measurements of 33–85 individual cells divided over three individual experimental replicated. Data shown as mean ± SEM. Significance was tested between the different metals (**A**, **B**) or between exposed and non-exposed cells (**D**–**F**) using a Mann-Whitney *U* test, where *p* < 0.05 is considered as statistically significant. The *p* values of significant results are indicated above a horizontal line between the tested conditions

It is known that metals, such as nickel [14], and to a lesser extent titanium [15], can increase the release of free radicals. However, to the best of our knowledge it is not known to date whether nitinol affects the free radical production in bronchial epithelial cells. The production of free radicals was measured using the T_1_ relaxometry mode of diamond magnetometry [22], where a lower T_1_ indicates increased production of free radicals. Using relaxometry T_1_ measurements, the levels of free radicals within a single cell can be assessed. To this end, the intracellular level of free radicals was measured in BEAS-2B cells that were in direct contact with a piece of nickel, titanium or nitinol for 4 or 48 h, as well as in cells that were only indirectly exposed to the metal pieces (Fig. 5A). In the cells that were not directly exposed to metal, no differences in free radical production were observed both at 4 and 48 h (Fig. 5B). In contrast, cells that were directly exposed to nitinol for 4 h displayed significantly lower levels of intracellular free radicals compared to cells directly exposed to nickel or titanium, as evidenced by a longer T_1_ time (Fig. 5C). After 48 h of direct exposure, the differences between the metals decreased, with only cells to exposed to nickel displaying significantly higher levels of intracellular free radicals compared to cells exposed to titanium (Fig. 5C). BEAS-2B cells directly exposed to a piece of nickel for 4 h displayed significantly higher intracellular free radical levels compared to cells not exposed to metal, while cells indirectly exposed to nickel displayed a trend towards higher intracellular free radical levels (Fig. 5D). After 48 h the free radical levels in the unexposed control cells increased to levels similar to cells exposed to a piece of nickel (Fig. 5D). For cells exposed to titanium a similar pattern was observed with a significant increase in free radical levels upon direct exposure to titanium for 4 h and a trend towards increased free radical for cells indirectly exposed to titanium, while these differences were absent after 48 h (Fig. 5E). For cells exposed to nitinol no increase upon either direct or indirect exposure for either 4 or 48 h was observed (Fig. 5F). Together, these data showed that BEAS-2B cells need to be in direct exposure to nickel or titanium in order to produce free radicals, and that nitinol does not induce increased production of free radicals, even upon direct exposure.

## Discussion

This is the first study that investigates the mechanisms involved in the interaction between nitinol and lung epithelial cells and fibroblasts. In the current study we showed that nitinol facilitates the growth of bronchial epithelial cells on its surface, while this was only sporadically observed for nickel or titanium. Furthermore, we showed that nitinol induces large differences in cellular activity, as the phosphorylation of several major cell signaling pathways was strongly affected by indirect exposure to nitinol. Although the phosphorylation response in our in vitro setting was rapid and short-lived, the in vivo response towards nitinol surfaces is likely to be more complex and pro-longed, involving secondary cells, such as neutrophils and macrophages. Lastly, we showed that especially in cells that are in direct contact with metal there is a strong induction of free radical production. Importantly, cells exposed to nitinol produce significantly less free radicals compared to cells exposed to nickel or titanium. Together, this study illustrates that the degree of cell proliferation as well as the activation of stress and inflammatory responses of lung epithelial cells and fibroblasts are dependent on the type of metal used for lung implantable devices. Although nitinol has strong effects on cellular signaling and may contribute to the initiation of inflammatory responses, it is generally better tolerated and less stress-inducing compared to nickel or titanium.

The implantation of devices within lung tissue induces stress, friction and injury to structural lung cells at the site of implantation. These physical pressure and stress cues, together with the chemical composition of the implanted materials and the chemicals and metal ions released from the implant surface, will be sensed by the lung structural cells. This may induce a foreign body response, where neutrophils and macrophages are attracted to the implant site, foreign body giant cells are formed and ultimately fibroblasts will be attracted, subsequently depositing extracellular matrix and starting the encapsulation of the implant [7]. When this process coincides with a chronic inflammatory trigger, granulation tissue is formed around the implantable device, often reducing the effectiveness of the device and initiating inflammation-related symptoms in the patients. Therefore, the capability of fibroblasts and epithelial cells to attach to and grow onto the implanted device is an important initial factor in the formation of fibrotic and granulation tissue. Nevertheless, some attachment of lung structural cells may be necessary to hold the implantable device at the right location and prevent device migration, another important risk factor for loss of implant effectiveness [2]. In our study it was shown that nitinol has a favorable surface to promote bronchial epithelial cell growth, as it was shown that cells form a monolayer on a nitinol surface within days. Fibroblasts on the other hand can attach and survive on a nitinol surface, but do not grow into a monolayer. These results are in agreement with previous studies showing that endothelial cells, as well as osteoblasts, are able to grow on nitinol surfaces, and that surface properties and the type of proteins adhered to the nitinol surface are important factors determining how well cells grow onto nitinol surfaces [24, 25].

In the current study we found limited inflammatory responses of lung epithelial cells and fibroblasts in response to metal surfaces. Therefore, this study was unable to identify the reason granulation tissue readily forms around lung implantable devices [26–28]. It may be that other cell types next to structural lung cells are needed to initiate an immune response, or that mechanical stimuli native to lung tissue such as stress and friction caused by breathing and coughing are needed. Additionally, the experimental model used in this study allows only a fraction of the cells to be directly exposed to metal, while most of the cells were indirectly exposed to metal, which may reduce the strength of the measured inflammatory signal. Additionally, the shape of the implantable device may also be important for the induced inflammatory response, as sharp edges may induce tissue damage. Therefore future studies should be performed using materials of different shapes. Nevertheless, this study showed an acute and strong effect of indirect nitinol exposure on cell signaling pathways in bronchial epithelial cells . After one hour of exposure to nitinol 18 out of the 39 measured cell signaling factors were increased, with STAT1, an important inducer of interferon-related inflammatory responses as well as a pro-survival factor, showing the highest increase with a 30 times increased expression [29, 30]. Although most of these cell signaling factors have complex and multi-facetted functions, most of the increased factors are involved in pro-inflammatory and cell proliferation processes. The increased activity of these factors seems temporarily, as after 72 h only STAT2 showed increased expression upon ongoing exposure to nitinol. The fact that even cells that are not directly exposed to nitinol display altered cell signaling may be caused by Ni^2+^ ions released from nitinol. It was previously reported that nickel ions are released from nitinol, and that these ions increase the expression of cell surface adhesion factors such as intercellular adhesion molecule 1, vascular cell adhesion molecule 1, and chemokine monocyte chemoattractant protein 1 [31]. We did not examine the expression of cell surface adhesion factors in our study, thus this would be an interesting topic for a follow-up study.

Oxidative stress caused by free radicals is one of the underlying mechanisms in metal toxicity. The biocompatibility of metals is related to the corrosion properties of the metal, and the chemical interaction between metal and host tissue [32, 33]. Although titanium is widely used as a biomaterial with a high success rate, there are rare reports of problems caused by released titanium particles. It was shown that titanium alloy particles were present in surrounding tissues due to corrosion and wear of implants, resulting in inflammatory responses and eventually loss of the initial treatment effect [34]. This is the first study to show that only cells directly exposed to metal display increased free radical production, while cells located in the proximity of the metal remain unaffected. We were able to assess this using a novel nanodiamond relaxometry-based method. This technique utilizes nano-sized diamonds containing NV centers, whose unique quantum properties allow them to be used for free radical sensing. The T_1_-relaxometry mode of diamond magnetometry uses the spin noise from unpaired electrons to detect free radicals [21–23]. This method is highly sensitive and specific for paramagnetic species. Additionally, we showed that cells exposed to nitinol do not display increased free radical production, demonstrating the superiority in biocompatibility of nitinol compared to nickel and titanium.

This study aimed to assess cellular and molecular responses of lung epithelial cells and fibroblasts that were either in direct contact with, or in the close proximity of metal. Adding a piece of metal to an already confluent layer of cells may trigger mechanosensors on the cells directly under the piece of metal, which may affect the experimental outcomes. Therefore, for most experiments in this study, the piece of metal was added to the cell culture well before the seeding of cells. This way, cells could grow and interact with the metal without differential forces being exerted by metals of different densities being placed upon the cell layer. Unfortunately, this technique was not possible when studying kinase phosphorylation profiles, as we aimed to study rapid effects that were induced immediately upon exposing cells to metal. To this end, we did carefully place a piece of metal onto the cell layer before studying the effect on kinase phosphorylation profiles induced by only one metal.

Together, our study indicates that the type of metal used for lung implantable devices may influence inflammatory and proliferative cellular responses in lung epithelial cells and fibroblasts. The data obtained from our study indicates that the material and surface properties of lung implantable devices influences the response of host cells to these implantable devices. Therefore, in order to ultimately prevent granulation and fibrotic tissue formation around implantable devices it may be necessary to apply specific coatings, or to alter the surface properties of the metal. Such approaches could be leveraged to reduce the formation of granulation tissue around implantable devices and increase the longevity and effectiveness of such implantable devices.

The main conclusions of our study are:

- Lung epithelial cells and fibroblasts respond differently upon exposure to different metals.

- Nitinol, and to a lesser extent nickel and titanium, surfaces support the attachment and growth of lung epithelial cells.

- Nitinol induces a rapid and significant alteration of kinase activity.

- Cells directly exposed to nickel or titanium produce free radicals, but those exposed to nitinol do not.

## References

[1] Gupta A, Burgess JK, Borghuis T, de Vries MP, Kuipers J, Permentier HP (2022). Identification of damage associated molecular patterns and extracellular matrix proteins as major constituents of the surface proteome of lung implantable silicone/nitinol devices. Acta Biomater.

[2] Roodenburg SA, Klooster K, Hartman JE, Koster TD, van Dijk M, Slebos D-J (2021). Revision bronchoscopy after endobronchial valve treatment for emphysema: indications, findings and outcomes. Int J Chron Obstruct Pulmon Dis.

[3] Mani G, Porter D, Grove K, Collins S, Ornberg A, Shulfer R (2022). Surface finishing of Nitinol for implantable medical devices: a review. J Biomed Mater Res B Appl Biomater.

[4] Guibert N, Saka H, Dutau H (2020). Airway stenting: Technological advancements and its role in interventional pulmonology. Respirology.

[5] van der Molen MC, Hartman JE, Vanfleteren LEGW, Kerstjens HAM, van Melle JP, Willems TP (2022). Reduction of lung hyperinflation improves cardiac preload, contractility, and output in emphysema: a clinical trial in patients who received endobronchial valves. Am J Respir Crit Care Med.

[6] Roodenburg SA, Hartman JE, Deslée G, Herth FJF, Klooster K, Sciurba FC (2022). Bronchoscopic lung volume reduction coil treatment for severe emphysema: a systematic review and meta-analysis of individual participant data. Respiration.

[7] Roodenburg SA, Pouwels SD, Slebos D-J. Airway granulation response to lung-implantable medical devices: a concise overview., Eur Respir Rev. 2021;30. 10.1183/16000617.0066-2021. 10.1183/16000617.0066-2021 PMC9488845 34348981

[8] McGrath DJ, Thiebes AL, Cornelissen CG, O’Shea MB, O’Brien B, Jockenhoevel S (2017). An ovine in vivo framework for tracheobronchial stent analysis. Biomech Model Mechanobiol.

[9] Fernández-Bussy S, Majid A, Caviedes I, Akindipe O, Baz M, Jantz M (2011). Treatment of airway complications following lung transplantation. Arch Bronconeumol.

[10] Thiebes AL, McGrath DJ, Kelly N, Sweeney CA, Kurtenbach K, Gesché VN (2019). Comparison of covered laser-cut and braided respiratory stents: from bench to pre-clinical testing. Ann Biomed Eng.

[11] Vrchovecká K, Mrázková J, Pávková Goldbergová M. Effect of Nitinol surface with nanotubes and/or ordered nanopores on cell behavior. Metallomics. 2022;14. 10.1093/mtomcs/mfac002. 10.1093/mtomcs/mfac002 35084501

[12] Duan W-X, He M-D, Mao L, Qian F-H, Li Y-M, Pi H-F (2015). NiO nanoparticles induce apoptosis through repressing SIRT1 in human bronchial epithelial cells. Toxicol Appl Pharmacol..

[13] Li Q, Suen T-C, Sun H, Arita A, Costa M (2009). Nickel compounds induce apoptosis in human bronchial epithelial Beas-2B cells by activation of c-Myc through ERK pathway. Toxicol Appl Pharmacol..

[14] Huang L, He F, Wu B (2022). Mechanism of effects of nickel or nickel compounds on intestinal mucosal barrier. Chemosphere.

[15] Zhang J, Shi J, Han S, Zheng P, Chen Z, Jia G (2022). Titanium dioxide nanoparticles induced reactive oxygen species (ROS) related changes of metabolomics signatures in human normal bronchial epithelial (BEAS-2B) cells. Toxicol Appl Pharmacol..

[16] Jacobs JP, Jones CM, Baille JP (1970). Characteristics of a human diploid cell designated MRC-5. Nature.

[17] Pouwels SD, Heijink IH, Brouwer U, Gras R, den Boef LE, Boezen HM (2015). Genetic variation associates with susceptibility for cigarette smoke-induced neutrophilia in mice. Am J Physiol Lung Cell Mol Physiol.

[18] Shenderova OA, Shames AI, Nunn NA, Torelli MD, Vlasov I, Zaitsev A (2019). Synthesis, properties, and applications of fluorescent diamond particles. J Vac Sci Technol B Nanotechnol Microelectron.

[19] Hemelaar SR, de Boer P, Chipaux M, Zuidema W, Hamoh T, Martinez FP (2017). Nanodiamonds as multi-purpose labels for microscopy. Sci Rep..

[20] Ong SY, van Harmelen RJJ, Norouzi N, Offens F, Venema IM, Habibi Najafi MB (2018). Interaction of nanodiamonds with bacteria. Nanoscale.

[21] Wu K, Vedelaar TA, Damle VG, Morita A, Mougnaud J, Reyes San Martin C (2022). Applying NV center-based quantum sensing to study intracellular free radical response upon viral infections. Redox Biol.

[22] Sharmin R, Hamoh T, Sigaeva A, Mzyk A, Damle VG, Morita A (2021). Fluorescent nanodiamonds for detecting free-radical generation in real time during shear stress in human umbilical vein endothelial cells. ACS Sens.

[23] Nie L, Nusantara AC, Damle VG, Baranov MV, Chipaux M, Reyes-San-Martin C (2022). Quantum sensing of free radicals in primary human dendritic cells. Nano Lett.

[24] Cattaneo G, Bräuner C, Siekmeyer G, Ding A, Bauer S, Wohlschlögel M (2019). In vitro investigation of chemical properties and biocompatibility of neurovascular braided implants. J Mater Sci Mater Med.

[25] Muhonen V, Fauveaux C, Olivera G, Vigneron P, Danilov A, Nagel M-D (2009). Fibronectin modulates osteoblast behavior on Nitinol. J Biomed Mater Res A.

[26] Koster TD, Klooster K, Ten Hacken NHT, van Dijk M, Slebos D-J (2020). Endobronchial valve therapy for severe emphysema: an overview of valve-related complications and its management. Expert Rev Respir Med.

[27] Mittal N, El-Said HG, Ratnayaka K, Rao A, Friesen TL, Nigro JJ (2021). Bronchial stenting in infants with severe bronchomalacia: technique and outcomes. Int J Pediatr Otorhinolaryngol.

[28] Aktaş Z, Öztürk A, Yılmaz A, Kızılgöz D, Yurtseven G (2019). Complications of silicone Y stents placed due to malignant airway stenosis. Tuberk Toraks.

[29] Tolomeo M, Cavalli A, Cascio A. STAT1 and its crucial role in the control of viral infections. Int J Mol Sci. 20222; 23. 10.3390/ijms23084095. 10.3390/ijms23084095 PMC9028532 35456913

[30] Rah B, Rather RA, Bhat GR, Baba AB, Mushtaq I, Farooq M (2022). JAK/STAT signaling: molecular targets, therapeutic opportunities, and limitations of targeted inhibitions in solid malignancies. Front Pharmacol..

[31] Li M, Yin T, Wang Y, Du F, Zou X, Gregersen H (2014). Study of biocompatibility of medical grade high nitrogen nickel-free austenitic stainless steel in vitro. Mater Sci Eng C Mater Biol Appl.

[32] Wever DJ, Veldhuizen AG, Sanders MM, Schakenraad JM, van Horn JR (1997). Cytotoxic, allergic and genotoxic activity of a nickel-titanium alloy. Biomaterials.

[33] Catelli E, Sciutto G, Prati S, Jia Y, Mazzeo R (2018). Characterization of outdoor bronze monument patinas: the potentialities of near-infrared spectroscopic analysis. Environ Sci Pollut Res Int..

[34] Kim KT, Eo MY, Nguyen TTH, Kim SM (2019). General review of titanium toxicity. Int J Implant Dent.

